# Effect of Simulated Matches on Post-Exercise Biochemical Parameters in Women’s Indoor and Beach Handball

**DOI:** 10.3390/ijerph17145046

**Published:** 2020-07-14

**Authors:** Joanna Kamińska, Tomasz Podgórski, Jakub Kryściak, Maciej Pawlak

**Affiliations:** Department of Physiology and Biochemistry, Poznań University of Physical Education, 61-871 Poznań, Poland; podgorski@awf.poznan.pl (T.P.); j.krysciak@awf.poznan.pl (J.K.); pawlak@awf.poznan.pl (M.P.)

**Keywords:** water-electrolyte status, acid-base balance, ambient condition, team sports, nutrition, women in sport

## Abstract

This study assesses the status of hydration and the acid-base balance in female handball players in the Polish Second League before and after simulated matches in both indoor (hall) and beach (outdoor) conditions. The values of biochemical indicators useful for describing water-electrolyte management, such as osmolality, hematocrit, aldosterone, sodium, potassium, calcium, chloride and magnesium, were determined in the players’ fingertip capillary blood. Furthermore, the blood parameters of the acid-base balance were analysed, including pH, standard base excess, lactate and bicarbonate ion concentration. Additionally, the pH and specific gravity of the players’ urine were determined. The level of significance was set at *p* < 0.05. It was found that both indoor and beach simulated matches caused post-exercise changes in the biochemical profiles of the players’ blood and urine in terms of water-electrolyte and acid-base balance. Interestingly, the location of a simulated match (indoors vs. beach) had a statistically significant effect on only two of the parameters measured post-exercise: concentration of calcium ions (lower indoors) and urine pH (lower on the beach). A single simulated game, regardless of its location, directly affected the acid-base balance and, to a smaller extent, the water-electrolyte balance, depending mostly on the time spent physically active during the match.

## 1. Introduction

Handball is a team sport played both indoors and outdoors, usually on the beach. In the first case, two teams of seven players each (including the goalkeeper) play a match that consists of two 30-min periods, on a solid floor court measuring 40 × 20 m. A beach handball match consists of two 10-min periods and is played by two teams with four players each (including the goalkeeper), on a sandy court measuring 27 × 12 m [1]. Matches are characterised by high-intensity movements (striding and sprinting) alternating with rest periods (walking, jogging and standing), which is referred to as stop-and-go [2,3].

Handball leads to high levels of perspiration, depending on the ambient temperature and humidity, as well as the individual player’s state of acclimatisation and physical fitness [4]. At the same time, significant individual variation in the amount of water lost through sweat is observed [5]. It is also known that water loss in excess of 2–3% of the player’s body mass causes a wide spectrum of disturbances, including thermoregulatory disorders [6], increase in the heart rate, decrease in plasma volume and in cardiac ejection fraction and nerve conduction disorders [7], which, in turn, reduce aerobic and anaerobic capacity and increase the likelihood of injury [1,8].

Sodium is eliminated through sweat to a greater extent compared to chlorides, potassium and other electrolytes [9]. The level of hydration has an influence the endocrine system, especially the synthesis of aldosterone, a hormone that helps maintain appropriate sodium levels in the body by increasing its reabsorption in the kidneys [10]. These processes and the resulting acidic compounds have an inevitable effect on the acid-base balance [11,12] leading to a number of adverse changes in the modulation of contractile proteins, the variability of the three-dimensional structure of proteins and even cognitive processes [13].

In sports practice, knowledge of blood and urine indicators of water-electrolyte and acid-base balance is seen as an important element in controlling training and match loads [14]. In the case of the handball players tested in this study, changes in the water-electrolyte and acid-base balance, in addition to purely environmental factors (indoors/beach), were also affected by the different types of surfaces. Movement on sand increases energy consumption compared to movement on a solid floor [15,16].

The aim of the study, in the absence of relevant data in the literature, was to describe changes to water-electrolyte and acid-base management caused by the adaptation of female handball players’ bodies to simulated matches, both indoors and on the beach. In addition, we investigated which of these workouts puts more pressure on the water-electrolyte and acid-base balance in the body.

Our hypothesis was that longer indoor matches would cause changes in both biological materials: peripheral blood and urine. Also, we hypothesised that the higher intensity of a match played on the beach, the higher energy cost of the effort made on sand and changing ambient conditions would cause deeper disturbances in the water-electrolyte and acid-base balance in the players’ bodies.

## 2. Materials and Methods

### 2.1. Experimental Approach

The changes in biochemical and haematological markers in female handball players during simulated indoor and outdoor (beach) matches were examined. The water-electrolyte balance and acid-base balance were assessed based on blood and urine parameters. The players participated in two matches played on an indoor solid floor court and two matches played on an artificial outdoor sand court in the transition period. Blood and urine samples were collected before and after each simulated match. The rationale behind these times of measurement was to assess the players’ hydration status on different surfaces and in different environmental conditions during a simulated match.

### 2.2. Participants

The investigation included 12 female athletes who played handball (AZS AWF Poznań—University Sports Association of Poznan University of Physical Education, Poland; Polish Second League) and trained indoors, out of which 6 participated in simulated matches on the beach in the summer period. Goalkeepers were excluded from the research due to different match efforts. The measurements were repeated for two successive years (2016 and 2017) with the same players. Because the indoor and the beach group was analysed over two years, this amounted to 24 and 12 cases, respectively.

The players’ anthropometric data were determined based on the average of three measurements made before the simulated matches in both years (Table 1). Body height and mass were measured using WPT60/150 OW medical scales (Radwag^®^, Radom, Poland), while waist circumference was measured using a tape measure. In addition, body mass was checked before and after each simulated match.

### 2.3. Ethics Approval 

All subjects gave their informed consent for inclusion before they participated in the study. The study was conducted in accordance with the Declaration of Helsinki, and the protocol was approved by the Ethics Committee of the Poznan University of Medical Sciences (Approval No.: 140/15).

### 2.4. Biochemical Analyses

The material for the tests was fingertip capillary blood obtained from each player’s non-dominant hand before and after the simulated matches. Blood was collected by qualified medical personnel in accordance with applicable procedures. The samples were drawn from the fingertip of the non-dominant hand using a disposable Medlance^®^ Red lancet-spike (HTL-Zone, Berlin, Germany) with a 1.5 mm blade and 2.0 mm penetration depth. Furthermore, a urine sample was obtained from each player.

The volume of 65 µL of blood was collected into a heparinised capillary tube, where the concentrations of sodium (Na^+^), potassium (K^+^), calcium (Ca^2+^), chloride (Cl^−^), bicarbonate (HCO_3_^−^) ions, blood lactate (La), blood pH value, standard base excess and osmolality were determined using a blood gas analyser (ABL90 FLEX, Radiometer, Copenhagen, Denmark). Moreover, 300 µL of capillary blood was collected into a Microvette^®^ CB 300 tube (Sarstedt, Nümbrect, Germany) containing K2-EDTA (EDTA dipotassium salt) as anticoagulant for haematological measurement using the 20-parametric automated haematology analyser Mythic^®^ 18 (Orphée, Geneva, Switzerland). Hematocrit value was considered in the study. Furthermore, 300 µL of capillary blood was collected into a Microvette^®^ CB 300 Z tube (Sarstedt, Nümbrect, Germany) with a clotting activator, and the separated serum was used to measure the concentration of aldosterone (DRG MedTek, Warsaw, Poland; Cat No. EIA-5298) and magnesium (Mg; Cormay, Łomianki, Poland; Cat No. 2-229), which was determined on a multi-detector microplate ELISA reader (Synergy 2 SIAFRT, BioTek, Winooski, Vermont, USA), whereas the specific gravity and pH of urine were measured with a device used for the biochemical analysis of urine (URYXXON^®^ Relax, Macherey-Nagel, Düeren, Germany).

### 2.5. Simulated Matches

**Figure 1:**
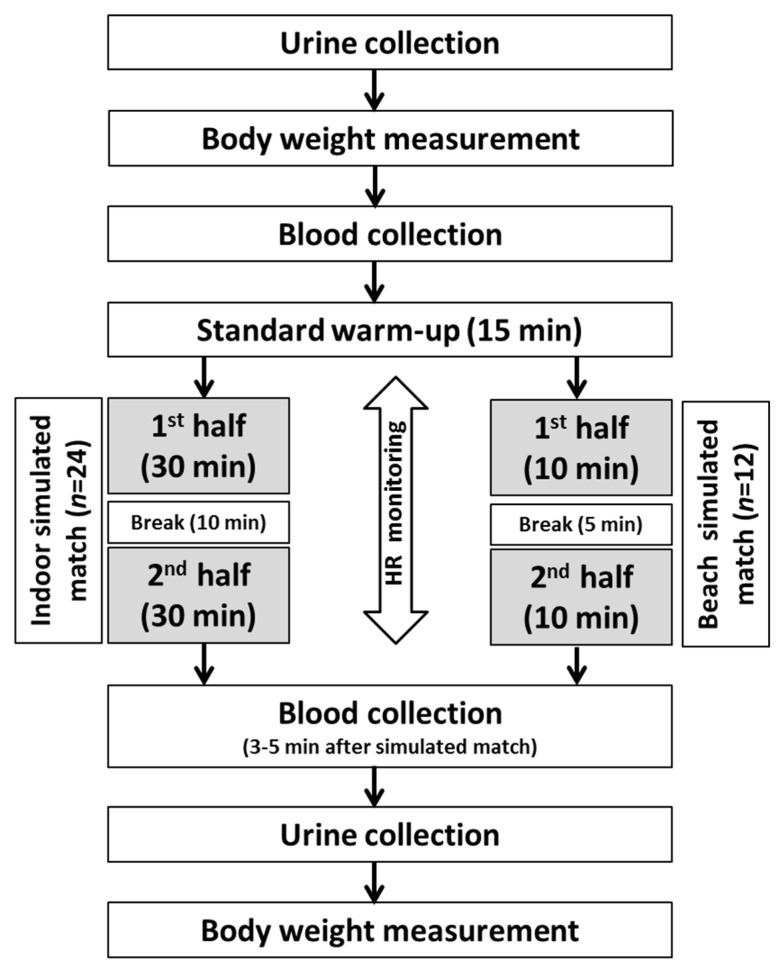
Pattern of testing for handball players playing indoors and on the beach.

Both the simulated indoor and beach matches took place in the same week, on Tuesday and Thursday, respectively, in the transition period between the last match of the indoor season and the start of the beach season (June). Testing was repeated for two successive years, according to a predetermined pattern (Figure 1). The simulated handball matches were played on an indoor solid floor court and an artificial outdoor sand court. Both courts were located in the same primary school which specialises in handball. In the four corners of court, temperature and humidity were measured using data loggers (EBI 310 TH, Ingolstadt, Germany) to determine ambient conditions. The mean measured temperature and humidity were significantly higher (*p* < 0.001) on the beach in comparison to indoors (beach vs. indoors: temperature 27.1 ± 0.83 °C vs. 21.0 ± 0.11 °C and humidity 53 ± 3.8% vs. 40 ± 2.6%, respectively). During the simulated matches, players declared maximum involvement in the game. The research assumed that players participated in the matches throughout the game. During each simulated match, each player’s heart rate (HR) was monitored with Polar Team^2^ PRO (Polar Electro Oy, Kempele, Finland) (Table 1). During the matches, the players themselves decided on the time and quantity (Table 1) of their intake of low-mineralized water (total mineral content = 231.9 mg/L) with the following mineral composition: Ca^2+^ (48.8 mg/L); Na^+^ (4.25 mg/L); Mg^2+^ (3.7 mg/L); K^+^ (0.47 mg/L); HCO_3_^−^ (136.0 mg/L); SO_4_^2−^ (17.0 mg/L); Cl^−^ (5.86 mg/L); F^−^ (0.13 mg/L).

### 2.6. Statistical Analysis

Data are presented as mean values with standard deviation ($\bar{X}$ ± SD) and confidence interval (95% CI). All measured variables were checked for normality of distribution using the Shapiro–Wilk test. In order to compare rest and post-exercise values between both match types, the homogeneity of variance of the individual indicators was examined, and the *t*-test for indicators with normal distribution and the Mann–Whitney U test for indicators with no normal distribution were carried out. In order to compare the differences between times (before and after the simulated matches), analysis of variance with repeated measures (ANOVA) and Wilcoxon signed-rank test were applied. Effect sizes (d) were calculated using means and standard deviations. To determine the effect size, Cohen’s criteria were used [17], which indicates that ≥0.2 and <0.5 was considered “small”, ≥0.5 and <0.8 “medium”, and ≥0.8 “large”. The level of significance was set at *p* < 0.05. The statistical analysis was performed using the analytics software package STATISTICA 13.1 (StatSoft Inc., Tulsa, OK, USA).

## 3. Results

The physiological homogeneity of the female athletes in the tested groups is demonstrated by the results of all variables measured before the simulated indoor and beach matches (Table 2). The results of these two groups were not statistically different. However, statistical differences were visible after the end of the match. An analysis of differences in the mean values of all the variables measured showed statistically significant differences for both mentioned locations in terms of: body mass, urine specific gravity, concentration of aldosterone, HCO_3_^−^, standard base excess, blood pH and lactate. Statistically significant post-exercise differences in the concentration of K^+^ and Ca^2+^ were found only in the case of indoor matches and were found in urine pH only on the beach. However, no significant differences were observed for hematocrit, osmolality and the concentration of Na^+^, Cl^−^ and magnesium (Table 2).

**Figure 2:**
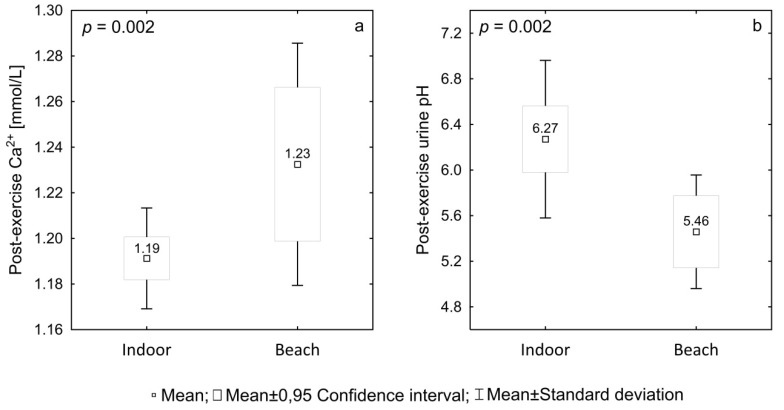
Significant post-exercise differences between simulated matches played indoors and on the beach: (**a**)—for Ca^2+^, (**b**)—urine pH.

The ambient conditions of simulated matches (indoor vs. beach) had a statistically significant effect on two parameters measured post-exercise: concentration of Ca^2+^ (Figure 2a) and urine pH (Figure 2b).

## 4. Discussion

The aim of this study was to compare the post-exercise response of female handball players caused by a simulated match in both beach and indoor conditions. We hypothesised that the longer indoor matches would cause changes in the players’ peripheral blood and urine. Also, we predicted that the higher intensity of a match on the beach, higher energy cost of the effort made on sand and changing ambient conditions would cause deeper disturbances in the water-electrolyte and acid-base balance in the players’ bodies.

To our knowledge, this is the first time that an extensive study describing post-workout changes in female handball players in terms of water-electrolyte and acid-base balance has been presented. In addition, this research considers the ambient conditions of simulated matches, played traditionally indoors in a hall or outdoors on a sand court. The value and practical usefulness of this work is evident, given the small number of publications on handball, especially regarding post-exercise response [18] as well as the characteristics of players’ hydration status [1].

In this study, a significant decrease in body mass amounting on average of 1.3 ± 0.46% was observed in all the players after the simulated matches. This was undoubtedly caused by insufficient fluid intake during these matches. However, the dehydration levels of the analysed participants did not exceed 2% of their respective body mass and, therefore, likely had little impact on their physical performance [1,8]. Previously, Cunniffe et al. [1] noticed that only 56% of female handball players took equal or greater volume of fluids than the amount of sweat expelled during training and competitive games. In other team sports, the reduction in body mass during training and during a match has been assessed at 0.5% and 0.6%, respectively, for female rugby players [19], and at 0.8% for male beach volleyball players during a match [20]. Higher post-exercise dehydration, reaching even −3.2% of body mass, has been observed in male basketball players during competitive games [21]. Male football players, in the publications of Maughan et al. [5] or Shirreffs et al. [22], showed dehydration levels exceeding 2% of their respective body mass, both after training and after a competitive game.

In the current work, a statistically non-significant reduction of Hct values were observed directly after the matches. Such an effect was also observed by Lippi et al. [23] after a half-marathon. However, some authors reported a decrease in Hct value after exercise [24,25], attributing this to post-exercise auto-hemodilution [26].

The proper hydration of the players before the simulated matches was confirmed by another indicator we examined, which is urine specific gravity. In most of them, this value was below 1.020 before the matches. When the value of this index reaches 1.021–1.030, it indicates that the body dehydrating in the range of 3% to 5% of body mass. Determining urine specific gravity has a practical dimension—athletes with higher values before an event of physical exertion risk demonstrating potentially greater dehydration after a training session or a match [21]. Osterberg et al. [21] recorded dehydration levels in basketball players immediately before a competitive game and then observed the exacerbation of this condition after the game.

A good marker for assessing the hydration of players is also plasma osmolality. Dehydration leads to a significant increase in plasma osmolality, for example after an ultramarathon [27] or high-volume training [28]. The present work did not show changes in the osmolality of plasma after simulated matches, which was probably compensated by the stable concentrations of blood sodium (Table 2) and glucose (data not shown).

Physical effort leads to loss of electrolytes through sweat [1,8,29]. In the present study, a statistically significant decrease of potassium and calcium concentrations in the players’ blood were observed, however, only after the indoor match. This not-fully-explainable physiological effect was also observed in a group of basketball players [30] and young boxers [31] immediately after training. Lowering calcium concentrations in blood plasma, as suggested by Wang et al. [30], could be caused by its transport to muscle and nervous tissue to help neural signalling, in response to physical effort.

Many factors may affect post-exercise potassium concentration in the blood. Some researchers have indicated that physical exercise does not significantly change the level of this marker [27,32], however, others have observed an increase [28] or even a reduction [28] thereof. The decrease of blood potassium concentration in this study may be due to regulation of the water-electrolyte balance by increasing aldosterone concentration. This steroid hormone preventing water loss through Na^+^ retention in the body may increase the excretion of potassium in the urine [28,29]. The current investigation showed a significant (*p* < 0.05) increase in aldosterone concentration, in both the simulated indoor and beach matches, which would confirm the existence of the mechanism described above.

Exercise usually leads to the loss of Na^+^, Cl^−^ and magnesium through sweat [1,33,34] that is not always combined with a simultaneous decrease of their concentration in blood [19,27,28,31], as was also confirmed in our study. Moreover, there are also reports showing an increase [28,30] as well as a decrease of blood sodium concentration after intense exercise [32,35]. Only a handful of authors have described post-exercise changes in blood chloride ion concentration, although the role of these ions during depolarization of skeletal muscle cells has been known for two decades. Wang et al. [30] reports their increase after high-intensity training in elite basketball athletes. Regarding post-exercise changes in magnesium concentration, Laires and Monteiro [36] showed that sub-maximal effort decreased, but lower intensity exercise increased, the concentration of blood magnesium ions. It can be assumed that the moderate-intensity exercise by the tested handball players did not have a sufficient trigger effect to disturb the homeostasis and change the concentration of these three electrolytes in their blood. Additionally, the lack of changes in their concentration in the blood may be explained by proper renal reabsorption and not very intense loss through sweat.

Disturbances in the acid-base balance of the body are manifested by changes in the blood lactate and bicarbonate ion concentrations, by disorders of both, value of the standard base excess, as well as the pH of blood and urine. In the current work, statistically significant post-exercise changes were found for all these indicators, with the lactate concentration increasing, while the values of the other indicators decreased. These changes indicate the acidification of the players’ plasma after both types of simulated matches, indoor and beach. The reduction of bicarbonate ion concentration after exercise is consistent with most published data [11,37], indicating the participation of these ions in buffering processes. Similar to HCO_3_^−^, a decrease in the standard base excess value is a typical response to intense physical exertion [11,38]. Both types of simulated matches caused aerobic and anaerobic metabolism (post-exercise lactate concentration below 4 mmol/L, Table 2), as a result of which the acid-base balance changed.

Female handball player experienced a reduction in blood and urine pH after physical exercise as a consequence of the acidic products of metabolism, such as hydrogen ions, lactate, pyruvate and ketone bodies. In the present study, the post-exercise results of this indicator, both in the blood and urine, were statistically significantly reduced. Most publications, according to the results of their own research, confirm a decrease in the value of blood pH after anaerobic [11], aerobic [12] and mixed physical exercise [39].

The influence of blood lactate in terms of lowering post-exercise pH was also shown in our study, supporting other studies’ observations of an increase in blood lactate concentration after exercise regardless of the nature of that exercise [11,12,37,39].

In this paper, we reported a few differences in biochemical parameters during the simulated matches in indoor and outdoor conditions. No papers presenting a comparison of urine and blood biochemical parameters between indoor and beach handball players have been published to date, to the best of our knowledge. It is worth noting that both temperature and humidity were significantly lower indoors compared to the beach, so this may have influenced the metabolic response of the players. A statistically significant decrease in the concentration of calcium ions observed only after the simulated indoor matches may be associated with the longer duration of this exercise. Decreases in muscle Ca^2+^ content and Ca^2+^-ATPase activity are closely related to depressed contraction possibilities caused by fatigue [40].

Shirreffs et al. [22] described changes in sweat loss depending on ambient conditions (temperature—T; humility—H). They determined that the percentage change in body mass of football players was the same in cool (T = 5 °C, H = 81%), moderate (T = 27 °C, H = 55%) and warm (T = 32 °C, H = 20%) environments. This shows that ambient conditions may not significantly affect sweat loss during training or during a match when players are able to adjust the amount of fluids, clothing and working rate [5], which is probably what happened in our study.

The strengths of this study include monitoring the hydration of athletes based on indicators verified in blood and urine samples. Furthermore, very few publications relating to team sports describe hydration in women, as most of them concern men. Unfortunately, this study did not include any monitoring of urine and sweat ions, nor an analysis of the athletes’ diet. Both could help document the influence of the foods consumed on the level of electrolytes and acid-base balance during the training process. In future studies, a higher number of analysed athletes should also be considered.

## 5. Conclusions

A simulated handball match, depending on the location where it was performed (indoor vs. the beach), directly affected the acid-base balance and, to a smaller extent, the water-electrolyte balance in the players.

The match effort in indoor handball had a higher impact on the electrolyte balance than that in beach handball, reducing blood calcium and potassium concentration. The intake of electrolytes in the form of liquids or gels by athletes playing or training indoors is more important than by athletes engaged in outdoor activity. This is probably due to the time spent physically active during these matches.

Due to a significant reduction in the concentration of potassium and calcium ions in the blood of the players who exercise in indoor conditions, it is recommended to use drinks with greater levels of mineralization than those used in this research.

## Figures and Tables

**Table 1:** Characteristics of female handball players taking part in the simulated indoor and beach matches.

Variable	Indoor (*n* = 24)		Beach (*n* = 12)		*p*-Value
	$\bar{X}\pm \mathrm{SD}$	95% CI	$\bar{X}\pm \mathrm{SD}$	95% CI	
Age (years)	21 ± 2	20–22	21 ± 2	19–22	NS
Body height (m)	1.70 ± 0.05	1.68–1.72	1.69 ± 0.05	1.66–1.72	NS
Body mass (kg)	63.2 ± 4.0	61.4–64.9	62.0 ± 4.3	59.2–64.7	NS
WHtR *	43.0 ± 2.6	41.9–44.1	43.2 ± 2.7	41.6–44.9	NS
HR mean (bpm)	151.5 ± 3.9	149.9–153.2	152.1 ± 2.6	150.5–153.8	NS
Fluids intake (mL)	561 ± 164	492–631	527 ± 162	424–630	NS

* waist-to-height ratio; NS—non-statistically significant.

**Table 2:** Average values of the analysed indicators measured before and after both types of simulated matches.

Indicator	Location	Pre-Exercise	Post-Exercise	*p*-Value	Effect Size
Body mass (kg)	Indoors	63.2 ± 4.0	62.2 ± 4.0	≤0.001	0.24
	Beach	62.0 ± 4.3	61.4 ± 4.3	≤0.001	0.14
**Water Management**					
Hematocrit (L/L)	Indoors	0.383 ± 0.020	0.378 ± 0.020	NS	
	Beach	0.381 ± 0.029	0.374 ± 0.023	NS	
Urine specific gravity	Indoors	1.016 ± 0.006	1.019 ± 0.004	0.013	0.58
	Beach	1.013 ± 0.004	1.019 ± 0.005	0.003	1.36
Osmolality (mOsm/kg)	Indoors	289.4 ± 2.4	289.5 ± 3.4	NS	
	Beach	289.5 ± 4.1	291.2 ± 4.2	NS	
**Electrolyte Management**					
Na^+^ (mmol/L)	Indoors	142 ± 1	142 ± 2	NS	
	Beach	142 ± 2	143 ± 2	NS	
K^+^ (mmol/L)	Indoors	4.3 ± 0.5	4.1 ± 0.4	0.046	0.48
	Beach	4.4 ± 0.4	4.4 ± 0.7	NS	
Ca^2+^ (mmol/L)	Indoors	1.21 ± 0.03	1.19 ± 0.02	≤0.001	0.96
	Beach	1.23 ± 0.04	1.23 ± 0.05	NS	
Cl^−^ (mmol/L)	Indoors	108 ± 2	107 ± 2	NS	
	Beach	107 ± 2	108 ± 2	NS	
Magnesium (mmol/L)	Indoors	0.89 ± 0.04	0.86 ± 0.08	NS	
	Beach	0.87 ± 0.02	0.85 ± 0.06	NS	
Aldosterone (mmol/L)	Indoors	124.6 ± 62.4	304.5 ± 168.9	≤0.001	1.41
	Beach	129.0 ± 102.1	213.7 ± 200.5	0.034	0.53
**Acid-base Balance**					
HCO_3_^−^ (mmol/L)	Indoors	24.6 ± 1.4	22.5 ± 1.8	≤0.001	1.28
	Beach	24.7 ± 1.7	23.4 ± 2.4	0.011	0.63
Standard base excess (mmol/L)	Indoors	0.3 ± 1.7	−2.5 ± 2.5	≤0.001	1.33
	Beach	0.3 ± 2.3	−1.5 ± 3.3	0.011	0.63
Blood pH	Indoors	7.42 ± 0.02	7.40 ± 0.03	0.002	0.90
	Beach	7.42 ± 0.02	7.40 ± 0.02	0.002	0.93
Urine pH	Indoors	6.21 ± 0.61	6.27 ± 0.69	NS	
	Beach	6.54 ± 0.62	5.46 ± 0.50	0.005	1.93
Blood lactate (mmol/L)	Indoors	1.26 ± 0.53	5.33 ± 3.01	≤0.001	1.89
	Beach	1.29 ± 0.41	5.75 ± 1.83	≤0.001	3.36

NS—non-statistically significant.
